# The natural course of nonculprit coronary artery lesions; analysis by serial quantitative coronary angiography

**DOI:** 10.1186/s12872-018-0870-9

**Published:** 2018-06-28

**Authors:** Jeehoon Kang, Kyung Woo Park, Michael S. Lee, Chengbin Zheng, Jung-Kyu Han, Han-Mo Yang, Hyun-Jae Kang, Bon-Kwon Koo, Hyo-Soo Kim

**Affiliations:** 1 Department of Internal Medicine and Cardiovascular Center, Seoul National University Hospital, 101 Daehakro, Jongno Gu, Seoul, 110-744 South Korea; 2 Division of Cardiology, University of California Los Angeles Medical Center, Los Angeles, CA USA

**Keywords:** Plaque progression, Nonculprit lesion, Coronary angiography, Quantitative coronary angiography

## Abstract

**Background:**

Nonculprit lesions are the major cause of future cardiovascular events. However, the natural course of nonculprit lesions and angiographic predictors of plaque progression are not well-studied. The purpose of our study was to observe the natural course of nonculprit lesions, and to identify predictors of unanticipated future events and angiographic progression in nonculprit lesions.

**Methods:**

We analyzed 640 nonculprit lesions with a length of ≥2 mm and luminal narrowing ≥30% from 320 patients who had two serial angiographic follow-ups; 9 to 13 months post-PCI and 24 months post-PCI. The study endpoints were nonculprit-ischemia driven revascularization (IDR) and the rate of diameter stenosis (DS) progression. Those with progression of DS > 12%/year were defined as ‘rapid progressors’.

**Results:**

During the median follow-up period of 737 days, 20 lesions in 20 patients (6.3%) required nonculprit-IDR. Independent predictors of nonculprit-IDR were diabetes (hazard ratio [HR] 2.93, 95% confidence interval [CI] 1.072–8.007, *p* = 0.036) and lesion type B2/C (HR 4.017, 95% CI 1.614–9.997, *p* = 0.003). The presence of one or both of the two major risk factors was associated with significant DS progression (3.0 ± 6.8% vs. 3.5 ± 6.1% vs. 6.8 ± 9.9% for lesions with 0, 1 and both risk factors, *p* < 0.001). Among the 640 lesions, 38 lesions (5.9%) in 33 patients were rapid progressors, while risk factors of rapid progressors included lesion type B2/C as a lesion-related risk factor (HR 1.998, 95% CI 1.006–3.791, *p* = 0.048) and diabetes mellitus as a patient-related risk factor (HR 3.725, 95% CI 1.937–7.538, p < 0.001). Lesions with both risk factors (type B2/C lesions in diabetic patients) were at the highest risk of rapid progression (odds ratio 3.250, 95% CI 1.451–7.282), compared to type A/B1 lesions in non-diabetic patients.

**Conclusion:**

Nonculprit-IDR was not uncommon during the 2-year follow up period in our population. The major risk factors of nonculprit lesion progression were diabetes and lesion type B2/C.

**Trial registration:**

Retrospectively registered and approved by the institutional review board of Seoul National University Hospital (No.: 1801–138-918) on February 2nd, 2018.

**Electronic supplementary material:**

The online version of this article (10.1186/s12872-018-0870-9) contains supplementary material, which is available to authorized users.

## Background

Nonculprit lesions account for a significant portion of future adverse cardiac events [1]. A previous study reported that nearly half of the major adverse cardiovascular events were associated with nonculprit lesions during a 3-year follow-up [2]. In the clinic, we can occasionally meet coronary artery disease patients with initially insignificant nonculprit lesions, which rapidly progress to cause ischemia despite standard medical treatment. These patients show a higher incidence of heart failure, recurrent acute coronary syndrome, leading to deterioration of the patients’ quality of life [3]. Recent studies have described a few mechanisms to explain the rapid progression in coronary stenosis, including coronary vasospasm [4], a complex stenosis morphology [5], and the systemic inflammatory status [6]. However, these mechanisms are putative, and studies on the progression of nonculprit lesions are limited. Therefore, efforts to detect the predictors of nonculprit lesion progression could prevent unanticipated future events related to these lesions.

Current studies of plaque progression focus on the rupture of vulnerable plaques, which present as myocardial infarction or cardiac death [7–9]. These studies mostly used intravascular ultrasound (IVUS) or optical coherence tomography (OCT) to evaluate plaque characteristics. However, IVUS and OCT are invasive techniques which are not routinely used to characterize insignificant lesions. In fact, clinical characteristics and coronary angiography, which is the most commonly used method to initially assess coronary lesions are the only information available [10].

In this study, we analyzed insignificant, non-treated lesions, performed a longitudinal analysis using serial quantitative coronary angiography (QCA). We observed the natural course of nonculprit lesions, and identified the predictors of unanticipated future events and angiographic progression in nonculprit lesions.

## Methods

### Study design and population

Coronary artery disease patients, who receive PCI in our institute, were enrolled in a stent registry at the time of the index procedure (i.e. Everolimus, Zotarolimus, or Biolimus eluting stent registry). According to the protocol of the individual registries, a follow up CAG was recommended (which is not mandatory) at 9 to 13 months post-PCI. Of those that agreed to and received the first follow up angiogram, a second routine follow up angiogram was recommended at 24 months. During the study period (July 2008 to March 2013), 3044 patients were enrolled in various stent registries, and 1486 patients (48.8%) received 9 to 13 month follow up CAG. Among these patients, 320 patients (21.9%) had a second routine follow up CAG at 24 months post-PCI. Patients with lesions of a lesion length of ≥2 mm and luminal narrowing ≥30% at baseline angiography were included in the analysis. As a sensitivity analysis to check the possibility of selection bias, the baseline demographics were compared with that of the entire 3044 patients that received PCI during the study period (Additional file 1: Table S2).

The study was approved by the ethics committee and institutional review board and was conducted according to the principles of the Declaration of Helsinki. All patients provided written, informed consent for participation in the registry.

### Quantitative coronary angiography

Coronary angiograms were recorded at baseline and at two serial follow-up periods. Analysis was done at the angiographic core laboratory by 3 specialized quantitative coronary angiography technicians at the Seoul National University Hospital Cardiovascular Clinical Research Center Angiographic Core Laboratory. Standard qualitative and quantitative analyses and definitions were used for angiographic analysis [11]. The ACC/AHA lesion classification system, comprising 11 angiographic variables with all lesions categorized into types (A, B1, B2 and C) were used to characterize lesions [12]. Measured variables included the reference vessel diameter, the minimal luminal diameter (MLD), and the diameter stenosis (DS). Delta DS was defined as the last DS minus the initial DS (DS at 1st follow-up when an adverse event occurred at the 1st follow-up, DS at 2nd follow-up when an adverse event occurred at the 2nd follow-up or in event-free cases.). Regarding reliability analysis, the intra-observer intraclass correlation (ICC) was 0.941 (95% CI 0.929–0.951) and the inter-observer ICC was 0.986 (95% CI 0.973–0.992).

### Study endpoints and definitions

The study endpoints were any event of nonculprit-ischemia driven revascularization (IDR) and the rate of DS progression during the follow-up period. Revascularization was defined as ischemia-driven if there was stenosis of at least 50% of the diameter with evidence of ischemia, as documented by a positive functional study, ischemic changes on an electrocardiogram, or ischemic symptoms. In the absence of documented ischemia, DS of at least 70% was required.

The definition of ‘rapid progressors’ was derived using the following method. A previous study reported that the rate of non-culprit lesion related clinical event was approximately 10% during 3 years of follow-up [2]. Therefore, we analyzed the cutoff point for DS progression in the upper 10% of the population using a histogram analysis. From this analysis ‘rapid progressors’ were defined as patients with at least one lesion that had DS progression more than 1% per month (or 12% per year). This is the rate of DS progression that would turn an average lesion in our study, into a significant one during the 24 month follow-up period.

### Statistical analysis

Continuous variables were presented as mean ± standard deviation and were compared using Student’s *t-*test or the Mann-Whitney U test. Categorical variables were presented as proportions, and chi-square test or Fisher’s exact test was applied to compare differences between groups, as appropriate. A comparison of baseline and follow-up values of the QCA results were analyzed by paired *t-*tests. To determine the independent predictors of nonculprit-IDR, a Cox proportional hazard model was used. Factors included into the multivariate model were lesion type, diameter of reference vessel, lesion location of the coronary artery as lesion-related factors, and age, gender, body mass index, diabetes, hypertension, chronic kidney disease, dyslipidemia, previous MI, smoking, and clinical diagnosis of ST segment elevation myocardial infarction as clinical factors. A multiple logistic generalized estimating equations (GEE) modeling using the autoregressive structure, was performed to analyze the longitudinal changes on lesion characteristics and clinical parameters as independent variables versus the progression of nonculprit lesions as the dependent variable. Variable included in the GEE model were identical to that included in the Cox proportional hazard model. Two-sided *P* values less than 0.05 were considered statistically significant for all tests. All statistical analyses were performed using SPSS version 20.0 (IBM Co., Chicago, IL, USA).

## Results

**Fig. 1:**
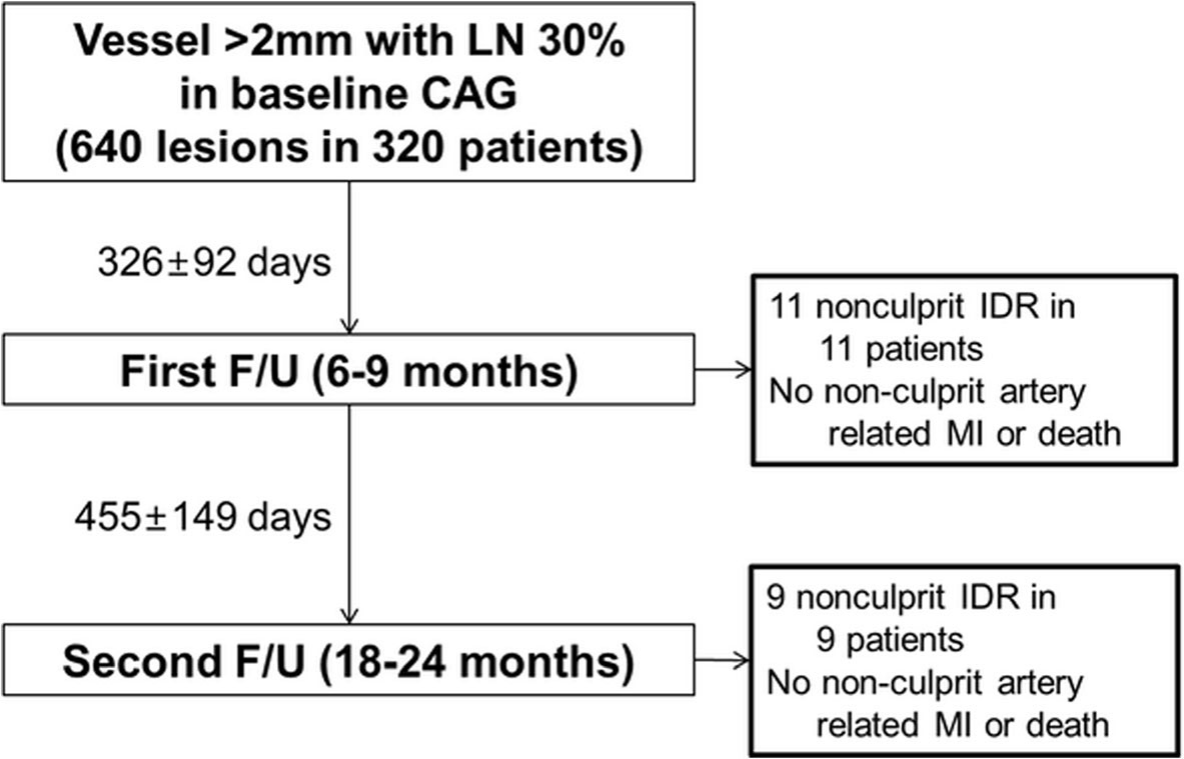
Study flow of the study

Between July, 2008, and March, 2013, a total of 320 patients with 640 lesions were enrolled in this study. Baseline demographic and clinical characteristics, initial laboratory findings and discharge medications of the study population are summarized in Additional file 1: Table S1. To show absence of selection bias of our study population, baseline demographics were compared with the total population whom received PCI during the study period (3044 patients, during July 2008 to March 2013; Additional file 1: Table S2) [13]. The mean follow-up duration from baseline to the first and second angiography was 326 ± 92 days and 759 ± 161 days respectively (Fig. 1). Laboratory findings at the follow-up periods are shown in Additional file 1: Table S3.

### Baseline lesion analysis and ischemia driven revascularization

Among the 640 baseline nonculprit lesions, 220 (34.4%) were located in the left anterior descending coronary artery, 181 (28.3%) in the left circumflex coronary artery, 237 (37.0%) in the right coronary artery, and 2 (0.3%) in the left main coronary artery. Also, 164 (25.6%) were proximal lesions, 152 (23.8%) were mid lesions, 171 (26.7%) were distal lesions and 153 (23.9%) were branches of epicardial coronary arteries. Baseline QCA revealed a lesion length of 11.4 ± 7.7 mm with a DS of 45.1 ± 10.5, 29.8% had a DS > 50, and 2.5% had a DS > 70%. There was no difference in lesion character in DM and non-DM patients (Additional file 1: Table S4).

The total follow-up duration was 640.3 patient-years. During the median follow-up period of 737 days, 20 lesions in 20 patients (6.3%) required nonculprit-IDR. Clinical and lesion characteristics between those with and without IDR are shown in Table 1. Patients who received nonculprit-IDR were more likely to have diabetes mellitus or chronic renal failure, and discharge medication pattern were similar between the two groups. Lesions that received IDR had a smaller MLD, larger DS, and were more likely to be B2/C lesions.

**Table 1:** Clinical and Lesion characteristic of lesions receiving ischemia-driven revascularization

Clinical Factors	IDR (+) (20 patients)	IDR (−) (300 patients)	*P* value
Age (years old)	62.5 ± 9.9	65.9 ± 10.3	0.152
BMI (kg/m^2^)	24.3 ± 3.2	24.8 ± 2.9	0.449
Gender (male), n (%)	16 (80.0)	223 (74.1)	0.557
Previous PCI, n (%)	2 (10.0)	35 (11.6)	0.825
Previous CABG, n (%)	1 (5.0)	3 (1.0)	0.119
Previous MI, n (%)	2 (10.0)	22 (7.3)	0.661
Previous CHF, n (%)	3 (1.0)	0 (0.0)	0.816
Diabetes mellitus, n (%)	12 (60.0)	96 (31.9)	0.010
Hypertension, n (%)	13 (65.0)	213 (70.8)	0.584
CRF, n (%)	2 (10.0)	7 (2.3)	0.044
Dyslipidemia, n (%)	9 (45.0)	190 (63.1)	0.106
Current smoking, n (%)	13 (65.0)	164 (55.2)	0.394
FHx of CAD, n (%)	1 (5.0)	35 (11.7)	0.610
Clinical diagnosis^a^, n (%)	60.0 / 25.0 / 5.0 / 10.0	64.7 / 18.7 / 10.7 / 6.0	0.616
Diagnosis of ACS, n (%)	8 (40.0)	106 (35.5)	0.688
LV ejection fraction (%)	61.2 ± 7.9	59.7 ± 8.8	0.482
WBC (10^9^/L)	7200 ± 2200	6800 ± 2300	0.550
Hemoglobin (g/dL)	13.8 ± 1.8	13.5 ± 1.8	0.485
Creatinine(mg/dL)	1.49 ± 1.43	1.08 ± 0.65	0.215
- eGFR (ml/min/1.73m^2^)	63.6 ± 28.4	72.9 ± 20.4	0.056
HbA1c (%)	7.2 ± 1.8	7.0 ± 1.0	0.693
Total cholesterol (mg/dl)	161 ± 31	157 ± 40	0.667
Triglyceride (mg/dl)	135 ± 56	136 ± 85	0.935
HDL-cholesterol (mg/dl)	44 ± 9	43 ± 11	0.585
LDL-cholesterol (mg/dl)	98 ± 31	97 ± 36	0.870
CRP (mg/dl)	0.41 ± 1.13	0.42 ± 1.23	0.981
Discharge Medications			
- Aspirin	20 (100%)	299 (99.7%)	0.796
- Clopidogrel	20 (100%)	299 (99.7%)	0.796
- Beta blocker	17 (85.0%)	213 (71.0%)	0.178
- ARB / ACE inhibitors	13 (65.0%)	140 (46.7%)	0.112
- Calcium channel blockers	5 (25.0%)	63 (21.0%)	0.672
- Statins	20 (100%)	296 (98.7%)	0.603
- High intensity statin	3 (15.0%)	77 (25.9%)	0.778
Lesion factors	IDR (+) (20 lesions)	IDR (−) (620 lesions)	P
Lesion length (mm)	16.14 ± 14.79	11.25 ± 7.35	0.157
Lesion location^b^, (%)	30.0/40.0/30.0/0.0	34.5/27.9/37.3/0.2	0.692
Lesion proximity^c^, (%)	20.0 / 15.0 / 30.0 / 30.0	26.5 / 24.8 / 27.5 / 24.5	0.703
Lesion type^d^, (%)	30.0 / 10.0 / 30.0 / 30.0	34.8 / 31.3 / 21.9 / 11.9	0.035
B2/C lesion type	12 (60.0%)	210 (33.9%)	0.016
Minimal lumen diameter (mm)	1.31 ± 0.49	1.59 ± 0.49	0.013
Reference diameter (mm)	2.86 ± 0.52	2.84 ± 0.57	0.868
Initial DS	54.3 ± 13.1%	44.8 ± 10.3%	< 0.001
Last DS	75.8 ± 14.8	47.9 ± 10.7%	< 0.001
Delta DS	22.1 ± 15.4%	3.1 ± 5.8%	< 0.001

*BMI* body mass index, *PCI* percutaneous coronary intervention, *MI* Myocardial infarction, *CABG* coronary artery bypass graft surgery, *CHF* Congestive heart failure, *FHx* family history, *CAD* coronary artery disease, *LV* left ventricle, *WBC* white blood cell, *HDL* high density lipoprotein cholesterol, *LDL* low density lipoprotein cholesterol, *CRP* C-reactive protein, *ARB* Angiotensin II receptor blockers, *ACE* Angiotensin-converting-enzyme, *DS* diameter stenosis

^a^Clinical diagnosis: Stable angina / Unstable angina / non ST-segment elevation myocardial infarction / ST-segment elevation myocardial infarction

^b^Lesion location: Left anterior descending artery / Left circumflex artery / Right coronary artery / Left main coronary artery

^c^Lesion proximity: Proximal lesion / Mid lesion / Distal lesion / Side branch

^d^Lesion type: Lesion type A / Lesion typeB1 / Lesion type B2 / Lesion type C

**Fig. 2:**
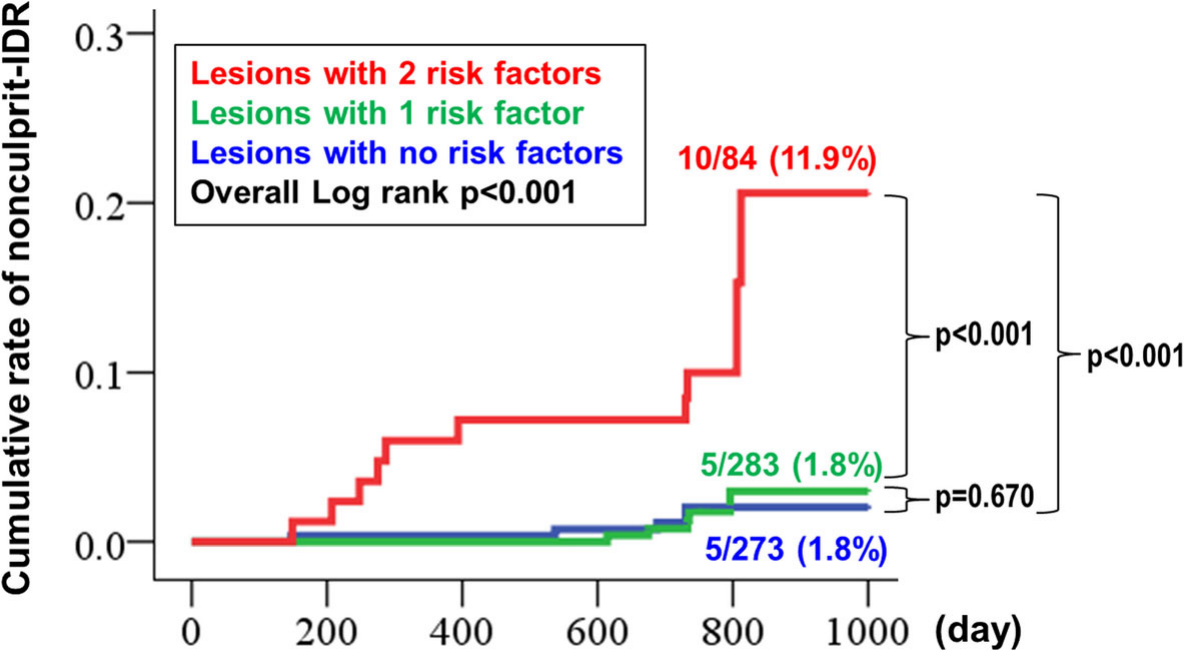
Survival curve of nonculprit-IDR. Nonculprit-IDR was significantly more frequent in lesions with both risk factors (diabetes and lesion type B2/C)

The independent baseline patient-level and lesion-level correlates of nonculprit-IDR were diabetes (hazard ratio [HR] 3.698, 95% confidence interval [CI] 1.377–9.933, *p* = 0.009) and lesion type B2/C (HR 3.510, 95% CI 1.376–8.955, p = 0.009), respectively. The rate of nonculprit-IDR for lesions increased along with the number of risk factors, showing a rate of 1.8, 1.8 and 11.9%, for lesions that included 0, 1, or both risk factors, respectively. Kaplan Meier curve of nonculprit-IDR events, according to the number of risk factors is shown in Fig. 2. The combination of these two risk factors increased the risk of IDR by at least 6-fold compared with any single risk factor.

### QCA analysis of the natural progression of atherosclerotic plaques

**Fig. 3:**
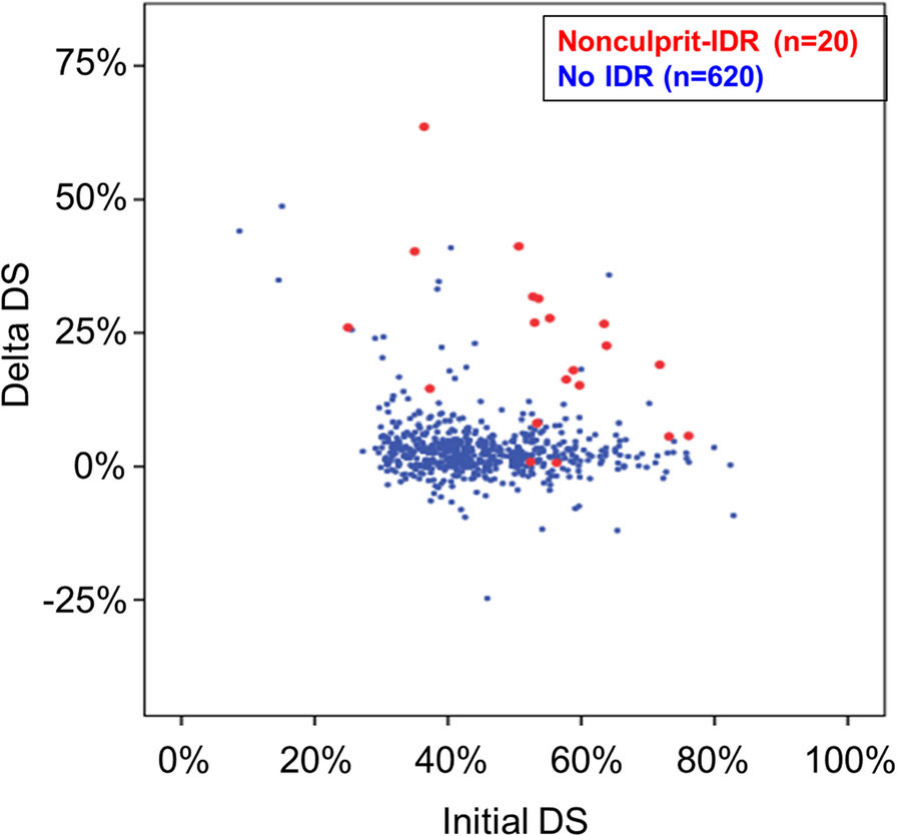
Dot plot of Initial DS and Delta DS

During the total follow-up period, the mean MLD decreased (1.58 ± 0.49 mm vs. 1.50 ± 0.48 mm vs. 1.46 ± 0.48 mm, *p* < 0.001, at baseline, 1st follow-up and 2nd follow-up, respectively) whereas the mean angiographic DS increased (45.1 ± 10.6 vs. 47.1 ± 11.2 vs. 48.2 ± 11.2, *p* < 0.001, at baseline, 1st follow-up and 2nd follow-up, respectively), suggesting continuous progression of angiographic stenosis (mean delta DS: 3.73 ± 7.09%) A dot plot of the baseline DS and delta DS of each lesion is shown in Fig. 3. Also, the velocity of DS progression was 2.46 ± 6.70%/year until the 1st follow-up, 1.49 ± 4.17%/year from the 1st follow-up to the 2nd follow-up period, and 2.19 ± 5.47%/year during the total follow-up period. A histogram of the velocity of all lesions is in Additional file 1: Figure S1. Lesions with nonculprit-IDR events had a larger delta DS (22.1 ± 15.4% vs. 3.1 ± 5.8%, *p* < 0.001) and faster rate of DS progression (21.12 ± 17.17%/year vs. 1.58 ± 3.14%/year, *p* < 0.001) compared to those without events.

### Predictors of progression

To investigate the predictors of DS progression, we used a GEE model for repeated measures. After correcting for the size and location of the coronary artery, male sex, low BMI (< 25 kg/m^2^), diabetes mellitus, and lesion type B2/C were significant risk factors for a higher DS. Among these factors, diabetes mellitus and lesion type B2/C lesions showed interaction with time during the follow-up period. In other words, patients with diabetes mellitus and type B2/C lesions showed faster progression of angiographic stenosis compared with those without these factors (Additional file 1: Table S5). The mean DS and mean delta DS according to the presence of 0, 1 or 2 risk factors are shown in Table 2. Compared with the angiographic stenosis progression of lesions with 0 or 1 risk factor, those with 2 risk factors had a significantly faster progression (delta DS of lesions with both risk factors: 2.3-fold larger compared to those with no risk factors and 1.9-fold larger compared to those with 1 risk factor). This was also consistent with comparison of the velocity of DS progression, where lesions with both risk factors showed a 2.8-fold larger velocity of DS progression.

**Table 2:** Initial and final Diameter Stenosis according to number of risk factors

	Group 1^a^	Group 2^a^	Group 3^a^	*P* value	
	No risk factors	1 risk factor	2 risk factors		
Initial DS	42.9 ± 9.6%	46.0 ± 11.0%	48.8 ± 10.5%	Overall	< 0.001
				Group 1 vs. Group 2	0.001
				Group 2 vs. Group 3	0.075
				Group 1 vs. Group 3	< 0.001
Final DS	46.0 ± 9.7%	49.5 ± 12.0%	55.5 ± 14.6%	Overall	< 0.001
				Group 1 vs. Group 2	0.001
				Group 2 vs. Group 3	< 0.001
				Group 1 vs. Group 3	< 0.001
Delta DS	3.0 ± 6.8%	3.5 ± 6.1%	6.8 ± 9.9%	Overall	< 0.001
				Group 1 vs. Group 2	0.703
				Group 2 vs. Group 3	0.001
				Group 1 vs. Group 3	< 0.001
Velocity of DS progression	0.150 ± 0.402%/month	0.154 ± 0.298%/month	0.422 ± 0.956%/month	Overall	< 0.001
				Group 1 vs. Group 2	0.995
				Group 2 vs. Group 3	< 0.001
				Group 1 vs. Group 3	< 0.001

^a^Group 1 implies those with diabetes mellitus (−) and B2C lesions (−). Group 2 implies diabetes mellitus (+), B2C lesions (−) or diabetes mellitus (−), B2C lesions (+) and Group 3 implies diabetes mellitus (+) & B2C lesions (+)

**Fig. 4:**
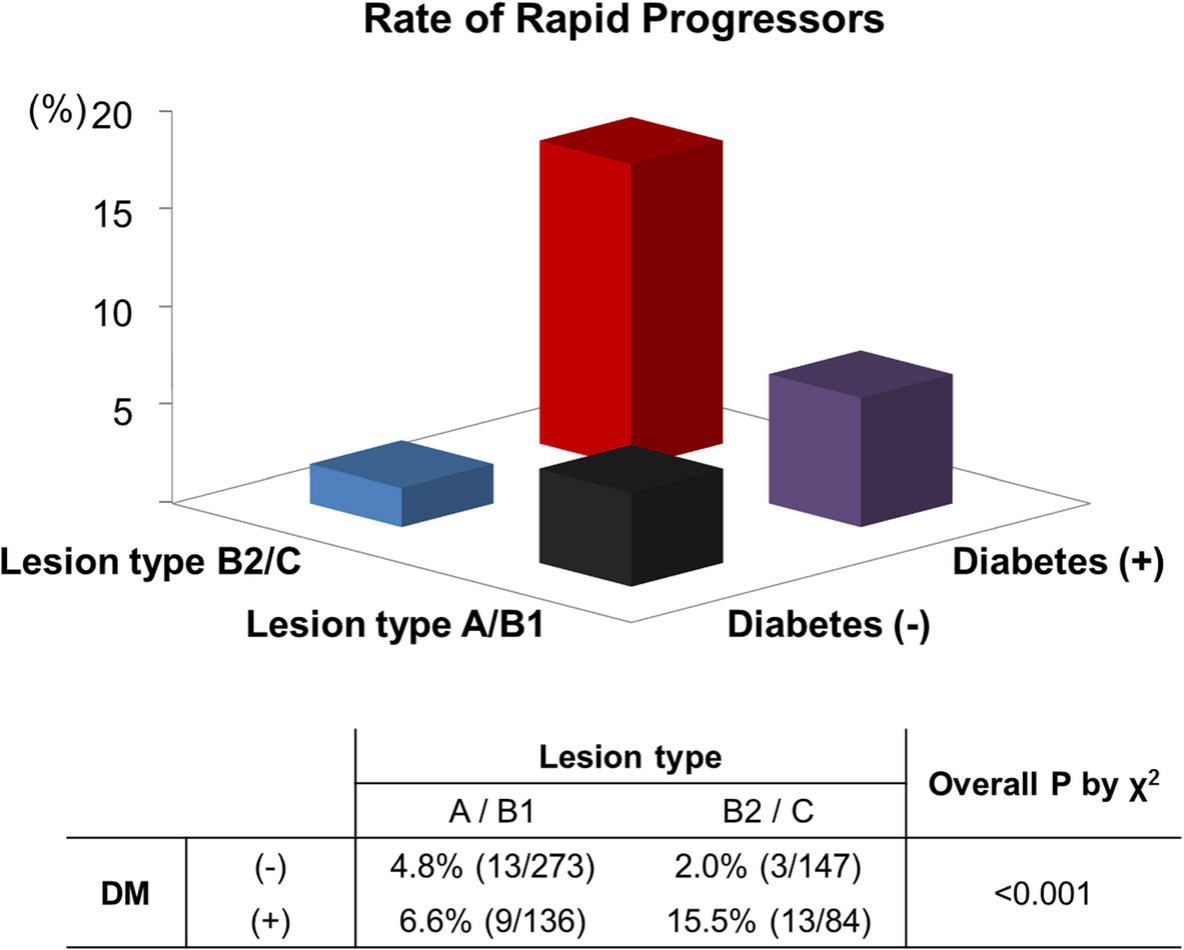
Rate of ‘Rapid Progressors’ by risk factor. Significantly more ‘Rapid progressors’ in lesions with both risk factors (diabetes and lesion type B2/C)

Among the 640 lesions, 38 lesions (5.9%) in 33 patients were defined as rapid progressors, defined as a progression of DS more than 1% year month or more than 12% per year. The GEE model for repeated measures revealed lesion type B2/C as a lesion-related risk factor for rapid progressors with a OR 2.139 (95% CI 1.066–4.294, *p* = 0.032) and diabetes mellitus as a patient-related risk factor (OR 2.782, 95% CI 1.349–5.737, *p* = 0.006). Lesions with both risk factors of diabetes and lesion type B2/C were at the highest risk of rapid progression (13/273 [4.8%] vs. 12/283 [4.0%] vs. 13/84 [15.5%], *p* < 0.001 for lesions with 0, 1, and 2 risk factors, respectively), with an odds ratio of 3.250 (95% CI, 1.451–7.282), compared to type A/B1 lesions in non-diabetic patients (Fig. 4).

## Discussion

In this study, the natural course of initially insignificant coronary artery lesions 6.3% of patients underwent nonculprit-IDR. Diabetes and lesion type B2/C were the major independent risk factors of nonculprit-IDR. The DS increased by 3.7 ± 7.1% (rate of 2.19 ± 5.47% per observed year) during the total follow-up period. The presence of both major risk factors resulted in a 2.8-fold faster DS progression compared to one or no risk factor. Rapid progression was found in 5.9% of lesions. Diabetes and lesion type B2/C were again the major predictors of rapid progression, and lesions with both risk factors showed a 3.25-fold higher risk of rapid progression, compared to those with no risk factors.

### Natural history of coronary plaques

The pathophysiology of coronary plaque formation has been studied for decades. Inflammation was identified as the primary driving force for activation and proliferation of smooth muscle cells, a processes mediated by growth factors. [14] Lesion progression can be induced by lipid accumulation, endothelial damage, or plaque erosion which involves many factors such as clinical risk factors of the individual, mechanical forces, etc. [15] Progression to a vulnerable plaque (known as thin cap fibroatheroma) [16], or injury such as deep plaque fissures or ulcerations, can cause acute coronary syndrome. [17]

Imaging techniques such as IVUS and OCT have been used to evaluate lesions and provide valuable information on plaque and lumen character. For example, the PROSPECT (Providing Regional Observations to Study Predictors of Events in the Coronary Tree) trial demonstrated that vulnerable plaques which are most likely to cause sudden unexpected adverse cardiac events can be pre-identified through IVUS imaging techniques. [2] In this study, the 3-year cumulative rate of major adverse cardiovascular events was 20.4%, among which, 11.6% were related to non-culprit lesions. Predictors for non-culprit lesions related events included insulin dependent diabetes and previous PCI as patient related factors, and a plaque burden of 70% or greater or a minimal luminal area of 4.0 mm^2^ or less or thin-cap fibroatheromas lesions as lesion related factors. In addition, the PROSPECT II trial is ongoing trial which will assess the ability of intracoronary near infrared spectroscopy (NIRS) to identify vulnerable plaques which subsequently lead to coronary events.

However, even these sophisticated imaging tools have shortcomings. For example invasive imaging tools cannot evaluate distal lesions, increases the time and expense of PCI and may cause coronary dissection or plaque erosion through physical contact. Also, evaluating the entire coronary vasculature with these tools is a significant clinical burden to both the clinician and patient. [18] Therefore, we still need more feasible angiographic standards for lesion evaluation.

In our study, we analyzed non-culprit lesions with serial coronary angiography and QCA, which permitted more detailed analysis. Compared to the PROSPECT trial, we could analyze the angiographic progression in every non-culprit lesion, including those which were not related to clinical events. We calculated the average angiographic progression rate in non-culprit lesions, the predictors of rapid progression, and also the predictors of clinical events. Regarding the fact that IVUS cannot be performed in every patient that we meet in the clinic, our results may give clinicians a more practical guideline for future events related with non-culprit lesions.

### Predictors of revascularization in nonculprit coronary plaques

Our population, in general, was receiving optimal medical therapy, with dual antiplatelet agents and lipid lowering agents. Previous studies have shown that statins could induce atheroma regression in coronary artery disease patients. [19, 20] Regarding lipid lowering agents, 93.8% were on statin, and the LDL level was very well-controlled, being 97 ± 35 mg/dL at the initial state and 67 ± 22 mg/dL and 65 ± 20 mg/dL at the 1st and 2nd follow-up respectively, suggesting that the study population, on average, were receiving appropriate statin therapy.

During the 2-year follow-up period, nonculprit-related events occurred in 6.3% of the patients, which were all revascularization events with no events of MI or nonculprit-related cardiac death. This was a similar rate to a previous study, where revascularization occurred in 10.5% during a 3-year follow-up. [2] Among lesion factors showing difference between those who and who did not experience nonculprit-IDR (i.e. smaller initial MLD, larger DS and had a higher proportion of lesion type B2/C), we were able to identify one lesion characteristic that was a significant predictor of subsequent events: lesion type B2/C. Diabetes was the only significant clinical factor to predict nonculprit IDR.

### Lesion progression and rapid progressors

Nonculprit lesions showed progression in DS at a velocity of approximately 2.2%/year. Factors associated with a faster progression over the follow-up period, were identical to those risk factors of nonculprit-IDR; diabetes and lesion type B2/C. It is well-known that local factors, such as tortuosity and irregular contours of the lesion (incorporated in the lesion type definition [12]) and systemic factors, such as diabetes both are related to lesion progression. [21, 22] From our result, lesions with risk factors had a 1.5-fold faster velocity of DS progression, compared to those without risk factors.

Among the total lesions, 5.9% were rapid progressors, with a progression of DS more than 1% year month or more than 12% per year. From the histogram of velocity of DS progression, there was a distinct group of lesions with faster progression. Predictors of rapid progression were identical to that of nonculprit-IDR (i.e. diabetes and lesion type B2/C); however there was a distinct pattern in the effect size of each risk factor. For predictors of nonculprit-IDR, lesion type B2/C had a larger effect size compared to diabetes, whereas predictors of rapid progressors, the effect size of diabetes was larger than that of lesion type B2/C. This can be partially explained by the character of the outcomes; nonculprit-IDR and rapid progressors. As nonculprit-IDR is outcome that embodies the discretion of the operator, high risk morphology of the coronary lesion, such as lesion type B2/C, could have influenced the procedure. On the other hand, rapid progressors was an angiographic finding which is free from any interference by the eye of the operator. Therefore, a more ‘ugly’ morphology of the nonculprit lesion, could have had more influence on nonculprit-IDR, compared to rapid progressors.

### Limitation

Although we performed a serial angiographic analysis of nonculprit lesion, our study has several important limitations. One inherent limitation is due to angiography itself. Even to the most experienced eye, angiography is a lumenogram yielding little insight into plaque composition or lesion pathology. It is well-known that plaques that show different composition have different outcomes, and that plaque composition does not always correlate with DS. Information regarding plaque character is something that is unobtainable from angiography. Also, other than morphology, fluid mechanics such as coronary wall shear stress is known to be associated with plaque progression, which was not analyzed. Second, due to the retrospective nature of our study, there could have been a selection bias in patient selection. We compared the baseline clinical characteristics with the total parent population whom received PCI during the study period, and we found minimal difference between the two populations. However, we cannot complete deny possibilities of other selection bias within our study population.

## Conclusion

Nonculprit-IDR was not an uncommon event during the 2-year follow up period in our population. Diabetes and lesion type B2/C are the major risk factors for nonculprit lesion IDR. Also, regarding angiographic progression, lesions with both risk factors showed a significantly rapid progression in DS compared to those with one or no risk factor.

## Additional file

Additional file 1: **Table S1.** Baseline demographic and clinical characteristics of the patients. **Table S2.** Baseline demographic and clinical characteristics of the patients. **Table S3. Table S4.** Lesion characteristic and initial QCA of the total lesions. **Table S5.** Initial and final Diameter Stenosis according to Diabetes and Lesion type. **Figure S1.** Histogram of DS progression and velocity of DS progression. (DOCX 204 kb)

## Abbreviations

CAG: Coronary angiography
CI: Confidence interval
DS: Diameter stenosis
GEE: Generalized estimating eqs.
HR: Hazard ratio
ICC: Intraclass correlation
IDR: Ischemia driven revascularization
IVUS: Intravascular ultrasound
MLD: Minimal luminal diameter
NIRS: Near infrared spectroscopy
OCT: Optical coherence tomography
OR: Odds ratio
PCI: Percutaneous coronary intervention
QCA: Quantitative coronary angiography
