# Supplementary material for: The natural course of nonculprit coronary artery lesions; analysis by serial quantitative coronary angiography
Source: BMC Cardiovasc Disord. 2018 Jun 28;18:130. doi: 10.1186/s12872-018-0870-9 (PMC6027760; doi:10.1186/s12872-018-0870-9)
Supplement: Supplementary file 1 — Table S1. Baseline demographic and clinical characteristics of the patients. Table S2. Baseline demographic and clinical characteristics of the patients. Table S3. Table S4. Lesion characteristic and initial QCA of the total lesions. Table S5. Initial and final Diameter Stenosis according to Diabetes and Lesion type. Figure S1. Histogram of DS progression and velocity of DS progression. (DOCX 204 kb) [file 12872_2018_870_MOESM1_ESM.docx]

**Additional file**

**The natural course of nonculprit coronary artery lesions; analysis**

**by serial Quantitative Coronary Angiography**

Jeehoon Kang et al.

1. **Supplementary Table**
2. **Supplementary Figure legends**

**1. Supplementary Tables**

**Table S1. Baseline demographic and clinical characteristics of the patients**

| Character | Value |
| --- | --- |
| Age (years old) | 65.7±10.3 |
| BMI (kg/m^2^) | 24.8±2.9 |
| Gender (male), n (%) | 239 (74.7%) |
| Previous PCI, n (%) | 37 (11.6%) |
| Previous CABG, n (%) | 4 (1.3%) |
| Previous MI, n (%) | 24 (7.5%) |
| Previous CHF, n (%) | 6 (1.8%) |
| Diabetes Mellitus, n (%) | 108 (33.8%) |
| Hypertension, n (%) | 225 (70.3%) |
| Chronic renal failure, n (%) | 9 (2.8%) |
| Dyslipidemia, n (%) | 198 (61.9%) |
| Smoking, (%)^*^ | 44.7 / 26.6 / 28.8 |
| FHx of CAD, n (%) | 36 (11.3%) |
| LV Ejection fraction, (%) | 59.8±8.8 |
| Clinical diagnosis, (%)^†^ | 63.4 / 19.1 / 10.3 / 6.3 |
| Number of Vessel disease, (%)^‡^ | 22.5 / 31.3 / 29.1 |
| Laboratory tests |  |
| WBC (10^9^/L) | 6850±2330 |
| Hemoglobin (g/dL) | 13.5±1.8 |
| Creatinine(mg/dL) | 1.10±0.73 |
| HbA1c (%, in diabetic patients) | 7.50±1.23 |
| - 9month HbA1c (%) | 6.90±0.93 |
| - 24month HbA1c (%) | 6.95±1.04 |
| Total cholesterol (mg/dL) | 157±39 |
| Triglyceride (mg/dL) | 136±84 |
| LDL (mg/dL) | 97±35 |
| HDL (mg/dL) | 43±11 |
| CRP(mg/L) | 0.42±1.22 |
| Discharge medication |  |
| Aspirin | 319 (99.7%) |
| Clopidogrel | 319 (99.7%) |
| Beta blocker | 228 (71.3%) |
| Statin | 300 (93.8%) |

BMI, body mass index; PCI, percutaneous coronary intervention; MI, Myocardial infarction; CABG, coronary artery bypass graft surgery; CHF, Congestive heart failure; FHx, family history; CAD, coronary artery disease; LV, left ventricle; WBC, white blood cell; HDL, high density lipoprotein cholesterol; LDL, low density lipoprotein cholesterol; CRP, C-reactive protein

* Smoking: Never smoker / Current smoker / Ex-smoker

^†^ Clinical diagnosis: Stable angina / Unstable angina / non ST-segment elevation myocardial infarction / ST-segment elevation myocardial infarction

^‡^ Vessel disease: 1 Vessel disease / 2 Vessel disease / 3 Vessel disease

**Table S2. Baseline demographic and clinical characteristics of the patients**

|  | Current study population | Total parent population | P value |
| --- | --- | --- | --- |
| Age (years old) | 65.7±10.3 | 65.0±10.3 | 0.278 |
| BMI (kg/m^2^) | 24.8±2.9 | 24.7±5.4 | 0.444 |
| Gender (male), n (%) | 239 (74.7%) | 2148 (70.6%) | 0.122 |
| Previous PCI, n (%) | 37 (11.6%) | 491 (16.2%) | 0.032 |
| Previous CHF, n (%) | 6 (1.8%) | 70 (2.3%) | 0.614 |
| Diabetes Mellitus, n (%) | 108 (33.8%) | 1170 (38.5%) | 0.096 |
| Hypertension, n (%) | 225 (70.3%) | 2058 (67.8%) | 0.361 |
| Chronic renal failure, n (%) | 9 (2.8%) | 181 (6.0%) | 0.020 |
| Current Smokers, (%) | 85 (26.6%) | 685 (22.5%) | 0.100 |
| FHx of CAD, n (%) | 36 (11.3%) | 385 (12.8%) | 0.443 |
| LV Ejection fraction, (%) | 59.8±8.8 | 58.7±9.1 | 0.052 |
| Clinical diagnosis |  |  | 0.066 |
| - Silent Ischemia | 18 (5.6%) | 115 (3.8%) |  |
| - Stable Angina | 188 (58.8%) | 1611 (53.0%) |  |
| - Unstable Angina | 61 (19.1%) | 683 (22.5%) |  |
| - NSTEMI | 33 (10.3%) | 360 (11.8%) |  |
| - STEMI | 20 (6.2%) | 272 (8.9%) |  |
| - Acute coronary syndrome | 53 (16.6%) | 632 (20.8%) | 0.075 |
| Vessel disease |  |  | 0.204 |
| - 1 Vessel disease, n (%) | 72 (27.2%) | 985 (32.5%) |  |
| - 2 Vessel disease, n (%) | 100 (37.7%) | 1048 (34.6%) |  |
| - 3 Vessel disease, n (%) | 93 (35.1%) | 1000 (33.0%) |  |

BMI, body mass index; PCI, percutaneous coronary intervention; CABG, coronary artery bypass graft surgery; CHF, Congestive heart failure; FHx, family history; LV, left ventricle; NSTEMI, non-ST segment elevation myocardial infarction; STEMI, ST segment elevation myocardial infarction

**Table S3.**

|  | baseline | 1^st^ follow-up | 2nd follow-up |
| --- | --- | --- | --- |
| WBC (10^9^/L) | 6850±2330 | 5980±1610 | 5910±1490 |
| Hemoglobin (g/dL) | 13.5±1.8 | 13.3±1.8 | 13.4±1.6 |
| Creatinine(mg/dL) | 1.10±0.73 | 1.03±0.77 | 1.08±1.06 |
| Total cholesterol (mg/dL) | 157±39 | 129±28 | 130±25 |
| Triglyceride (mg/dL) | 136±84 | 117±53 | 113±57 |
| LDL (mg/dL) | 43±11 | 45±12 | 47±12 |
| HDL (mg/dL) | 97±35 | 67±22 | 65±20 |
| CRP(mg/L) | 0.42±1.22 | 0.14±0.48 | 0.21±0.75 |

WBC, white blood cell; HDL, high density lipoprotein cholesterol; LDL, low density lipoprotein cholesterol; CRP, C-reactive protein

**Table S4. Lesion characteristic and initial QCA of the total lesions**

|  | Total population | DM (-) | DM (+) | p |
| --- | --- | --- | --- | --- |
| Lesion length | 11.40±7.71mm | 11.15±7.39 | 11.87±8.29 | 0.265 |
| Lesion location*, (%) | 34.2 / 27.3 / 37.0 | 34.8 / 26.7 / 38.1 | 33.6 / 31.4 / 35.0 | 0.457 |
| Lesion proximity^†^, (%) | 33.7 / 31.2 / 35.1 | 33.8 / 30.2 / 36.0 | 33.5 / 33.0 / 33.5 | 0.785 |
| Lesion type^‡^, (%) | 34.7 / 29.2 / 23.4 / 12.7 | 33.8 / 32.6 / 20.7 / 12.9 | 36.4 / 26.9 / 25.0 / 11.8 | 0.365 |
| Minimal lumen diameter | 1.58±0.49mm | 1.60±0.51 mm | 1.53±0.46 mm | 0.063 |
| Reference diameter | 2.84±0.56mm | 2.86±0.60 mm | 2.80±0.50 mm | 0.146 |
| Diameter Stenosis | 45.08±10.55% | 44.63±10.19 % | 45.95±11.17 % | 0.143 |

* Lesion location: Left anterior descending / Left circumflex / Right coronary artery

^†^ Lesion proximity: Proximal lesion / Mid lesion / Distal lesion

^‡^ Lesion type: Lesion type A / Lesion typeB1 / Lesion type B2 / Lesion type C

**Table S5. Initial and final Diameter Stenosis according to Diabetes and Lesion type**

| Risk factor | DM(-) | DM (+) | P value |
| --- | --- | --- | --- |
| Initial DS | 44.6±10.2% | 46.0±11.2% | 0.143 |
| Final DS | 47.7±10.7% | 50.9±13.7% | 0.003 |
| Delta DS | 3.1±6.1% | 5.0±8.6% | 0.004 |
| Velocity of  DS progression | 0.15±0.35 %/month | 0.27±0.66 %/month | 0.012 |
| Risk factor | **Lesion type A/B1** | **Lesion type B2/C** | **P value** |
| Initial DS | 43.7±10.2% | 47.7±10.6% | <0.001 |
| Final DS | 47.0±10.8% | 52.2±13.0% | <0.001 |
| Delta DS | 3.3±7.0% | 4.5±7.2% | 0.049 |
| Velocity of  DS progression | 0.16±0.38 %/month | 0.24±0.63 %/month | 0.032 |

**2. Supplementary Figure Legends**

**Figure S1.** Histogram of DS progression and velocity of DS progression

(A)


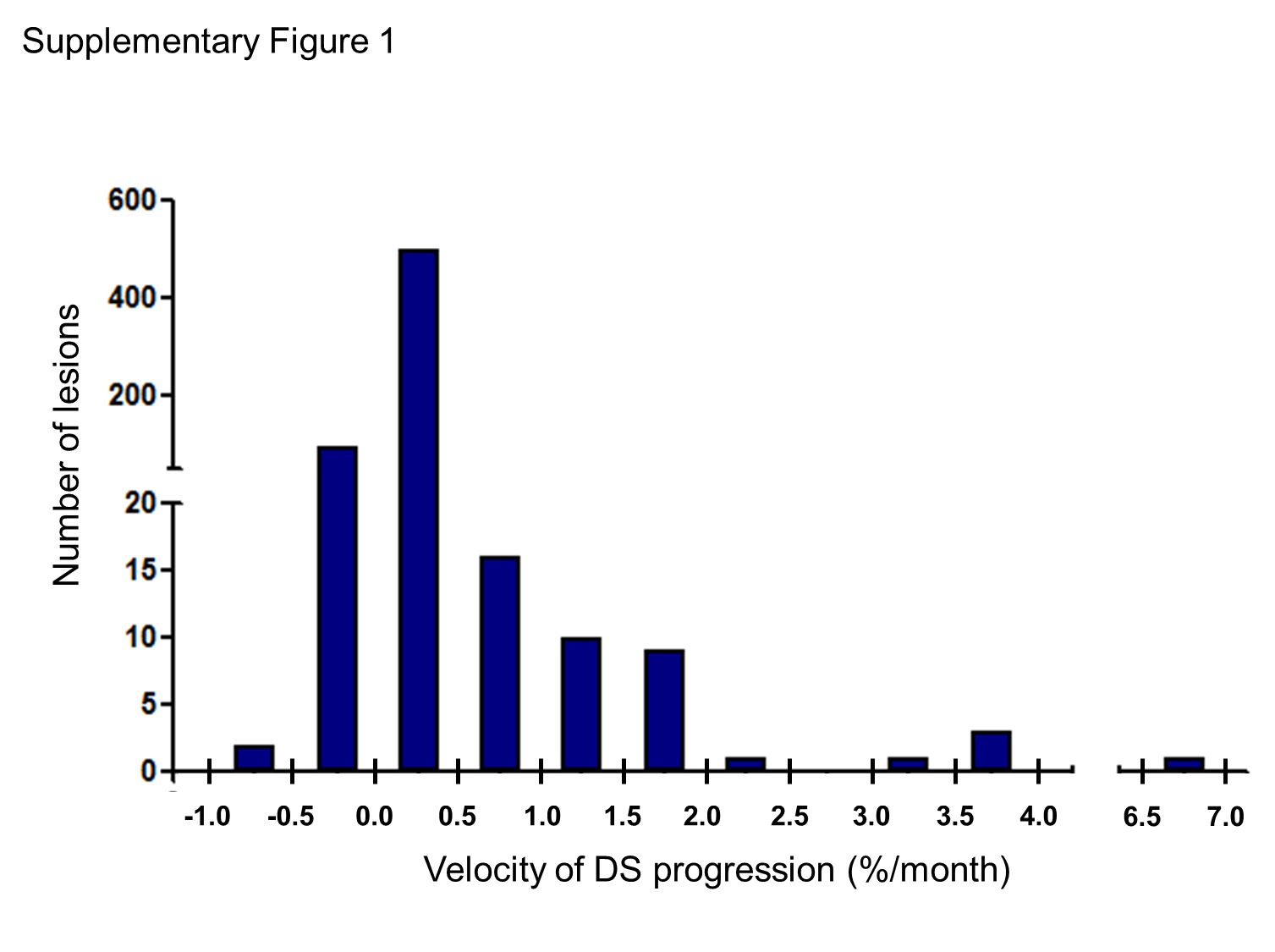


(B)


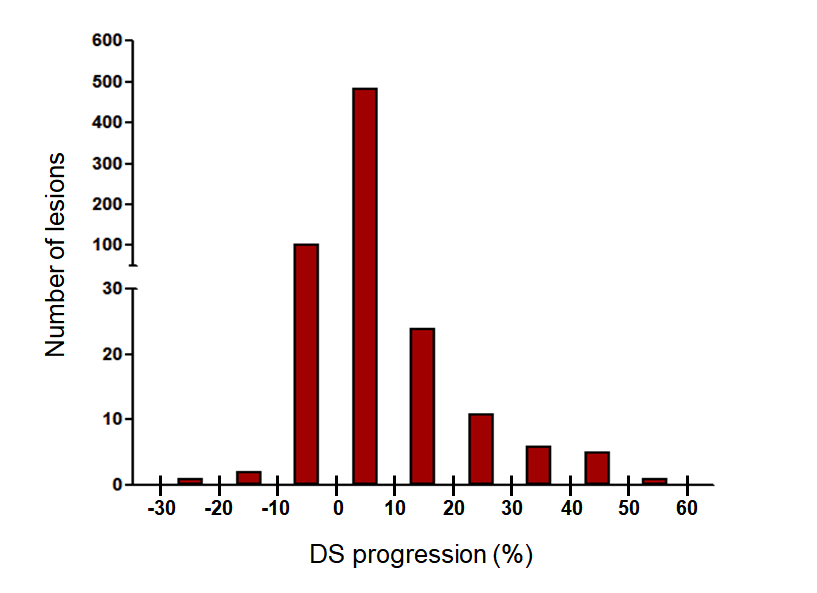


(A) The DS progression was 3.73% ± 7.09 %, with a range of -24.7% to 63.6%. (B) The velocity of DS progression was 0.19 ± 0.50 %/month, with a range from -0.92 %/month to 6.67 %/month. Among the total population, 108 lesions (16.9%) in 88 patients (27.5%) showed a negative value of DS progression (denoting plaque regression), among which 12 lesions (1.9%) in 10 patients (3.1%) had a DS progression value less than -5.0% (denoting a plaque regression more than 5%). Also, 532 lesions (83.1%) inn 232 patients (72.5%) had a positive value of DS progression (denoting plaque progression), among which 143 lesions (22.3%) in 122 patients (38.1%) had a DS progression value larger than 5.0% (denoting a plaque progression more than 5%).
